# Comparison of High-Dose versus Low-Dose Trimethoprim–Sulfamethoxazole for Treating *Pneumocystis jirovecii* Pneumonia among Hemodialysis Patients: A Nationwide Database Study in Japan

**DOI:** 10.3390/jcm13185463

**Published:** 2024-09-14

**Authors:** Hisayuki Shuto, Shota Omori, Kazufumi Hiramatsu, Jun-ichi Kadota, Kiyohide Fushimi, Kosaku Komiya

**Affiliations:** 1 Respiratory Medicine and Infectious Diseases, Faculty of Medicine, Oita University, 1-1 Idaigaoka, Hasama-machi, Yufu 879-5593, Oita, Japan; shuto0326@oita-u.ac.jp (H.S.);; 2 Research Center for Global and Local Infectious Diseases, Faculty of Medicine, Oita University, 1-1 Idaigaoka, Hasama-machi, Yufu 879-5593, Oita, Japan; 3 Department of Health Policy and Informatics, Tokyo Medical and Dental University Graduate School, 1-5-45 Yushima, Bunkyo-ku 113-8519, Tokyo, Japan

**Keywords:** trimethoprim–sulfamethoxazole, hemodialysis, *Pneumocystis jirovecii* pneumonia, cohort study, mortality

## Abstract

**Background:** Hemodialysis patients are at high risk for developing *Pneumocystis jirovecii* pneumonia (PJP), and trimethoprim–sulfamethoxazole (TMP–SMX) is the first-line agent for treating this disease. However, there is a lack of consensus on the required dosage of TMP–SMX for hemodialysis patients. **Methods:** This study used the nationwide Japanese Diagnosis Procedure Combination database to review hemodialysis patients hospitalized for PJP from April 2014 to March 2022. Eligible patients were divided into high-dose and low-dose groups based on the median daily dose per body weight of TMP. The 90-day mortality and adverse events after propensity score matching were compared between the groups. **Results:** A total of 126 hemodialysis patients with PJP were included, and the median daily dose per body weight of TMP was 5.74 mg/kg/day (interquartile range: 4.33–8.18 mg/kg/day). Thirty-two pairs were analyzed after the propensity score matching. No significant differences in the 90-day mortality and proportion of adverse events were observed between the high-dose and low-dose groups. **Conclusions:** A high dose of TMP–SMX is unlikely to decrease the in-hospital mortality and adverse events among hemodialysis patients with PJP. However, the results should be interpreted with caution, given the lack of power and lack of long-term follow-up. Additional prospective interventional studies are required to validate these results.

## 1. Introduction

*Pneumocystis jirovecii* pneumonia (PJP) is a significant life-threatening infectious disease that can occur in individuals with immunosuppressed conditions [1,2]. *Pneumocystis jirovecii* is a fungus that spreads via the airborne route and is found only in human alveoli. Humans experience *Pneumocystis jirovecii* infections during early childhood following the colonization of the fungus within the lungs of healthy individuals; the infected individuals are usually asymptomatic and develop severe pneumonia when complicated by immunodeficiency [3,4]. The risk factors for developing PJP include congenital or acquired immunodeficiency, such as human immunodeficiency virus (HIV) infection and iatrogenic immunosuppression, especially in organ transplant recipients, patients with autoimmune diseases, and individuals undergoing chemotherapy for malignant tumors [2]. Patients with end-stage renal failure are also at a higher risk of developing PJP, and hemodialysis patients have an approximately 30-fold increased risk of developing this disease [5]. Although the mechanisms for the increased risk of PCP in dialysis patients have not been clearly revealed, it is thought to result from combined immunodeficiency involving decreased CD4-positive cell function [6].

Trimethoprim–sulfamethoxazole (TMP–SMX) inhibits the synthesis of tetrahydrofolate acid, which is necessary for bacterial DNA synthesis and is used to treat urinary tract, respiratory, gastrointestinal, and skin infections [7]. Pentamidine was historically the first-line treatment for PJP; however, it was replaced by TMP–SMX due to its higher therapeutic efficacy [8]. The standard dose for patients with normal renal function is 15–20 mg/kg/day (the TMP component) [9,10,11,12]. This dose was determined based on a clinical study conducted on 20 pediatric patients with leukemia or sarcoma who developed PJP; survival superiority was observed in patients treated with 20 mg/kg/day of TMP compared with those treated with 4–7 mg/kg/day [13]. The maximum concentrations of the dosing interval (Cmax) in the high-dose group ranged from 5 to 8 mcg/mL of TMP [13]. Therefore, subsequent clinical studies were conducted using this value as a favorable blood level [14,15,16].

However, approximately 10–20% of TMP and 20% of SMX are metabolized in the liver and excreted, especially from the kidneys [14]; thus, a reduction in the dose is required to treat PJP in patients with renal dysfunction. Only a few studies on the pharmacokinetics of TMP–SMX in hemodialysis patients have been published in the literature [17]. Nissenson et al. [18] administered TMP–SMX (160 mg/day TMP) to 16 hemodialysis patients and reported clearances of 44% (TMP) and 57% (SMX) after 4 h of hemodialysis. Based on these findings, a TMP dose of 160 mg/day or 5 mg/kg/day has been recommended for treating PJP in dialysis patients [19,20,21,22]. However, the TMP–SMX clearance measured by Nissenson et al. [18] was elucidated in hemodialysis patients who did not develop PJP. Therefore, the appropriate dosage required to improve the prognosis of hemodialysis patients with PJP remains unclear.

In 2013, a single case report showed that favorable drug blood levels (5–8 mcg/mL of TMP) [13] were observed when 15 mg/kg/day of TMP was administered to a patient that underwent extended daily dialysis [23]. The authors suggested that the dosage recommended by Nissenson et al. [18] might be too low to treat PJP among patients undergoing hemodialysis using highly efficient dialysis membranes. However, the results of the case report cannot be directly applied to all patients receiving hemodialysis. To our knowledge, no additional studies on TMP–SMX dosing for dialysis patients who develop PJP during hemodialysis have been published in the literature. Clinical practice guidelines or global standards for the appropriate dosage of TMP–SMX for patients with PJP have not been established. Therefore, this study aimed to determine whether high or low doses of TMP–SMX could improve the prognosis of dialysis patients who received treatment for PJP.

## 2. Materials and Methods

### 2.1. Data Source

This nationwide database study was conducted using the Diagnosis Procedure Combination (DPC) inpatient database. With more than 1700 acute care hospitals in Japan adopting the DPC system, it covers data on most acute care patients in Japan [24]. This database contains information on age, sex, body mass index (BMI), scoring of activities of daily living (the Barthel index), comorbidities, smoking history (Brickman Index), disturbance of consciousness (Japan Coma Scale) on admission, primary diagnoses, complications, procedures, prescriptions, length of hospital stay, and discharge status. The diagnoses were recorded using the International Classification of Diseases, 10th Revision (ICD-10) codes, and the Japanese disease names [25].

This study was approved by the Institutional Ethics Committee of Oita University Faculty of Medicine (approval no. 2762, 22 March 2024). All aspects of the study complied with the Helsinki Declaration. The need for informed consent was waived because of the retrospective nature of the study, and information on the study was posted on the institutional website using the opt-out method.

### 2.2. Patients

Patients over 18 years old admitted to hospitals using the DPC system between April 2014 and March 2022 who developed PJP during hemodialysis (intermittent renal replacement therapy [IRRT]) and were undergoing treatment with oral or intravenous TMP–SMX were included in this study. Patients with ICD-10 codes B59 or J173, which are assigned to PJP, were selected from the nationwide DPC dataset in Japan. PJP was previously assigned the ICD-10 diagnosis code J17.3, which is a part of J17 for pneumonia, and is currently assigned to B59 in the classification of protozoan diseases. Thus, both B59 and J173 are used in the Japanese DPC system. The DPC database does not contain the diagnostic methods. The exclusion criteria were as follows: no confirmed diagnosis of PJP; not treated with PJP or treated with PJP for less than 7 days; TMP–SMX was not initiated during hemodialysis; hemodialysis was performed with continuous renal replacement therapy (CRRT); PJP treatment was initiated with drugs other than TMP/SMX, such as atovaquone or pentamidine; treated with <160 mg of TMP/day, which made it difficult to distinguish from prophylactic administration [11]; HIV complications; and missing weight data.

### 2.3. Data Collection and Outcomes

Background data, including age, sex, BMI, smoking history, comorbidities, Barthel index, and state of consciousness when admitted to the hospital, were collected. The Charlson comorbidity index was used to assess the comorbidities [26]. Information about hypoxia, mechanical ventilation, or cytomegalovirus co-infection within 7 days of TMP–SMX initiation during the PJP treatment was documented. Coinfection with cytomegalovirus was determined based on the use of anticytomegalovirus agents, including ganciclovir or valganciclovir. Information about the dose of TMP–SMX, duration of administration, dose reduction from the initial dose, switching from TMP–SMX to other drugs, and use of combination agents for PJP was collected. Treatment agents other than TMP–SMX included atovaquone [27], caspofungin [28] in combination with TMP–SMX, dapsone [29], pentamidine [30], primaquine in combination with clindamycin [31], and corticosteroids [32]. Steroid pulse therapy was defined by a maximum daily corticosteroid dose of 100 mg/day or more of methylprednisolone equivalent.

The eligible patients were divided into high-dose and low-dose TMP–SMX groups based on the median daily dose per body weight of TMP (due to a lack of specific definitions for the doses), and the outcomes were compared between the two groups. The primary endpoints were all-cause in-hospital mortality within 90 days and survival length after the initiation of PJP treatment. The secondary endpoints included tolerance to TMP–SMX, such as duration of administration, dose reduction from the initial dose, switching to other treatments, and adverse events. Although many types of adverse events have been reported for TMP–SMX [14,33,34], the DPC database does not include medical record articles written by physicians, and thus, fails to provide information for specific types of adverse events. Therefore, information on the following three adverse events was collected based on the available surrogate information for treatments in the database: hyponatremia, hypoglycemia, and thrombocytopenia. These were identified by the use of hypertonic sodium infusion, hypertonic glucose solution, and platelet transfusion, respectively.

### 2.4. Statistical Analysis

Statistical analyses were performed using IBM SPSS version 26 software (IBM, Armonk, NY, USA). A *p*-value of <0.05 was considered statistically significant. The two groups were compared using the *t*-test for continuous variables and the chi-square or Fisher’s exact test for categorical variables. Propensity score matching was performed to adjust for baseline patient backgrounds, clinical variables associated with disease severity, and treatment options other than TMP–SMX therapy. Logistic regression analysis was used to select the factors for calculating the propensity score. The caliper width was set at 20% of the standard deviation of the propensity score. Kaplan–Meier curves were constructed, and the log-rank test was performed to compare the survival lengths of the high-dose and low-dose TMP–SMX groups. Univariate analyses were performed using Cox hazard analysis to determine the association between in-hospital death within 90 days and other factors, including the use of high-dose TMP–SMX.

## 3. Results

### 3.1. Patient Characteristics

**Figure 1:**
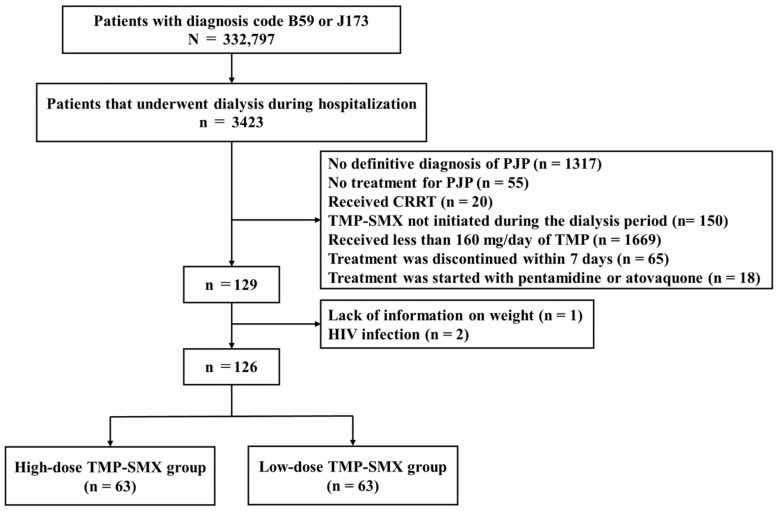
The dosage was divided into high or low based on the median (5.74 mg/kg/day) TMP–SMX dose among the 126 patients.

Of the 332,797 patients with a B59 (n = 262,656) or J173 (n = 70,569) diagnosis code, including overlap coding cases (n = 428), 3423 underwent renal replacement therapy during hospitalization. Among them, 1317 patients with no definitive diagnosis of PJP, 55 patients who were not treated for PJP, and 20 patients who received CRRT were excluded from this study. Of the remaining 2031 patients, 150 did not receive TMP–SMX during hemodialysis; 1669 received <160 mg/day of TMP; 65 presented with discontinuation of the PJP treatment within 7 days; and 18 were treated with other regimens, such as pentamidine or atovaquone, and were excluded from the study. Additionally, two patients with HIV and one lacking data on weight were excluded. Finally, 126 patients with PJP were eligible for this study (Figure 1).

All eligible patients received hemodialysis three times a week or once every two days. The median daily TMP dose per body weight was 5.74 mg/kg/day (interquartile range [IQR]: 4.33–8.18 mg/kg/day). TMP–SMX was administered orally (n = 118 [oral only, n = 96]), intravenously (n = 30 [iv only, n = 8]), or via both routes (n = 22). The patients were divided into two groups based on the TMP–SMX dose: high dose and low dose, with the cutoff value. In total, 31% (39/126) of patients were females, and the median age was 71.0 years (IQR: 64.0–78.0 years). As shown in Table 1, 75% and 14% of patients received oxygen or mechanical ventilation within 7 days from the start of treatment, respectively. Corticosteroids were administered to 91% of the patients; among them, 25% (29/114) were treated with steroid pulse therapy (Supplementary Table S1). Comparisons between the nonsurvivor and survivor groups in the hospital within 90 days revealed that the nonsurvivor group was older and had a lower Barthel index; moreover, a larger proportion of the patients in the nonsurvivor group received oxygen supplementation, ventilator management, and steroid pulse therapy within 7 days of starting the PJP treatment. The doses of TMP–SMX were significantly higher in the nonsurvivor group than in the survivor group.

### 3.2. Propensity Score Matching

The body weight and BMI in the high-dose group were significantly lower than those in the low-dose group. Furthermore, a tendency toward high doses of TMP–SMX for patients with preserved activities of daily living on admission, such as those with a high Barthel index and no impaired consciousness, and for those with severe PJP who received ventilator support or steroid pulse within 7 days of starting TMP–SMX, was observed (Table 2). A propensity score was calculated from these variables, except for BMI because of the overlap with body weight. As shown in Supplementary Figure S1, the area under the curve was estimated to be moderately high (0.763). After propensity score matching, 36 patients were selected from the 63 patients in each group; no significant differences in characteristics were observed between the high- and low-dose groups, except for the missing data on the smoking status (Table 2).

### 3.3. TMP–SMX and 90-Day Mortality in the Hospital

**Figure 2:**
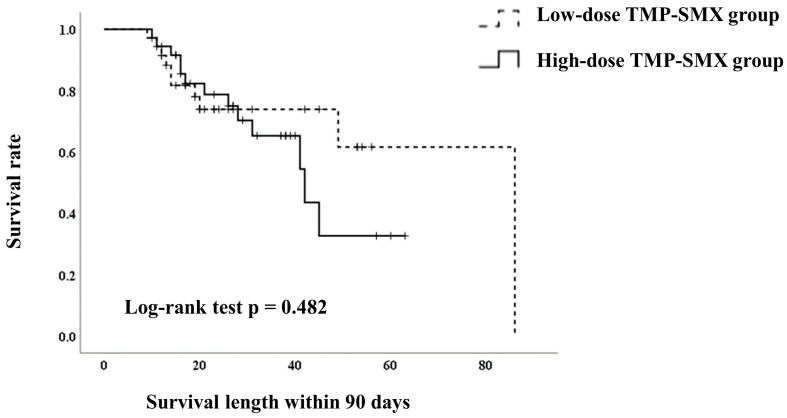
Kaplan–Meier curves showing the survival lengths of patients in the high-dose and low-dose TMP–SMX groups after propensity score matching.

The 90-day mortality rate in the high-dose TMP–SMX group was 36% (13/36), while that in the low-dose TMP–SMX group was 28% (10/36, Table 3). The survival lengths were not significantly different based on the log-rank test (Figure 2) (*p* = 0.482). The Cox hazard analysis also showed no significant association between high-dose TMP–SMX and 90-day mortality (hazard ratio [HR], 1.358; 95% confidence interval [CI], 0.575–3.210; *p* = 0.485). Ventilator support within the 7 days of treatment was significantly associated with the 90-day mortality (HR, 7.859; 95% CI, 3.182–19.410; *p* < 0.001; Table 4).

### 3.4. Tolerance and Adverse Events of TMP–SMX

After the propensity score matching, the median TMP doses were 8.50 mg/kg/day (IQR: 7.18–9.55) in the high-dose group and 4.28 mg/kg/day (IQR: 3.25–4.98) in the low-dose group (Table 3). The durations of TMP–SMX administration between both groups were comparable before and after the propensity score analysis. In the crude comparison, 39 adverse events (29 patients) were observed in the high-dose group and 30 (26 patients) in the low-dose group; however, the incidences between the groups were comparable after the propensity score analysis.

## 4. Discussion

The aim of this study was to determine whether high or low doses of TMP–SMX could positively affect the prognosis of dialysis patients who received treatment for PJP. No statistically significant association was observed between the two doses and the 90-day in-hospital mortality among the hemodialysis patients who developed PJP using the propensity-score-matching methods. Systematic reviews of observational studies showed that the efficacy of reduced-dose regimens is preserved during the treatment of PJP in nondialysis patients [35,36]. Furthermore, a phase III trial (LOW-TMP trial, NCT 04851015) comparing the efficacy and safety of low-dose TMP–SMX (10 mg/kg/day of TMP) with the standard dose TMP–SMX for nondialysis patients is currently ongoing [37]. Given the results from the current study in dialysis patients and the recent reviews in nondialysis patients, high-dose TMP–SMX regimens may not be superior to low-dose regimens for treating PJP in patients with or without hemodialysis. This may be because low doses of TMP–SMX can reach the appropriate levels required for killing *Pneumocystis jirovecii*, as suggested by several previous studies [19,20,21,22].

However, our study may not have completely ruled out selection biases despite the application of the propensity-score-matching method. A high-dose of TMP–SMX tends to be administered to critically ill patients on ventilator support. The efficacy of the high-dose TMP–SMX may be underestimated due to unknown confounding factors, such as the attending physician’s knowledge or beliefs, because the available medical data in the DPC database is limited. Furthermore, while the duration of TMP–SMX administration in the high-dose TMP–SMX group was comparable with that in the low-dose group after propensity score matching, many patients tended to switch to other drugs in the unmatched high-dose group (Table 3). This result suggests that high-dose TMP–SMX may be less tolerable over the long term [14]; nonetheless, details of the adverse effects could not be obtained due to a lack of data.

The Cox hazard analysis after propensity score matching showed that only ventilator use was significantly associated with 90-day mortality (Table 4). This result aligns with those of a previous systematic review, in which respiratory failure was associated with a poor prognosis [38]. In general, advanced age, female sex, respiratory failure, and solid tumors have been identified as risk factors for mortality among patients with PJP [39]. The current study found no other prognostic factors, probably due to the small number of cases.

The main strength of this study was the analyzed data on PJP treatment in patients undergoing hemodialysis using the nationwide database. However, our study had some limitations, in addition to the already mentioned selection biases. First, we could not access the information on laboratory data, including the blood levels of the drugs and the detailed conditions of hemodialysis, such as the membranes and flow. Even with the same dose per body weight, drug blood levels may differ in individuals due to variations in the drug excretion capacity that depend on the dialysis conditions [18,23]. It remains unclear whether low doses of TMP–SMX can reach the appropriate levels required for killing *Pneumocystis jirovecii*. Additional studies based on blood levels are inherently needed to elucidate the appropriate dosage of TMP–SMX for PJP treatment. Second, the number of eligible cases after the propensity score analysis was small, which resulted in a lower evaluation power of the outcomes. Thus, this study might provide statistical significance between the low-dose and high-dose groups. Third, other clinical outcomes, including rehospitalization and PJP relapse, could not be evaluated using the database. Finally, besides TMP–SMX, pentamidine and atovaquone may be used as first-line agents in treating PJP in hemodialysis patients [27,30,40]. Selection bias may have occurred because patients who were susceptible to side effects with TMP–SMX may have been treated with drugs other than TMP–SMX.

In conclusion, this study compared the 90-day hospital mortality and adverse effects, with a focus on hyponatremia, hypoglycemia, and thrombocytopenia, between high-dose and low-dose TMP–SMX using a national dataset of hemodialysis patients with PJP. No significant associations were found between the two groups. A high dose of TMP–SMX might not lead to a favorable clinical outcome. A prospective interventional study referring to the results of the ongoing trial and focusing on low-dose regimens for patients with normal renal function in the phase III trial is warranted to determine the appropriate TMP–SMX dosage in dialysis patients.

## Figures and Tables

**Table 1:** Baseline characteristics of the hemodialysis patients with PJP in the survivor and nonsurvivor groups (within 90 days of treatment).

	All Patients (n = 126)	Nonsurvivor (n = 40)	Survivor (n = 86)	*p*-Value
Sex: female	39 (31)	12 (30)	27 (31)	0.875
Age, years	71.0 (64.0–78.0)	74.5 (69.0–79.0)	70.0 (62.0–77.0)	0.001
BMI, kg/m^2^	20.9 (18.7–23.6)	21.1 (18.7–23.5)	20.9 (18.4–23.6)	0.778
Missing data	4 (3)	0 (0)	4 (5)	0.306
Weight	52.9 (46.1–61.1)	51.8 (48.9–59.7)	53.2 (45.0–61.9)	0.322
Barthel index	85.0 (25.0–100.0)	65.0 (7.5–100.0)	90.0 (55.0–100.0)	0.044
0–24	25 (20)	12 (30)	13 (15)	0.051
25–49	4 (3)	1 (3)	3 (3)	1.000
50–79	18 (14)	6 (15)	12 (14)	0.876
80–100	58 (46)	14 (35)	44 (51)	0.090
Missing data	21 (17)	7 (18)	14 (16)	0.864
Smoker	47 (37)	13 (33)	34 (40)	0.246
Missing data	19 (15)	4 (10)	15 (17)	0.277
Charlson comorbidity index *	2.0 (1.0–3.0)	2.0 (1.0–3.0)	2.0 (1.0–3.0)	0.959
0	10 (8)	4 (10)	6 (7)	0.724
1–2	69 (55)	19 (48)	50 (58)	0.264
3–4	35 (28)	14 (35)	21 (24)	0.217
5–6	6 (5)	1 (3)	5 (6)	0.664
7–9	6 (5)	2 (5)	4 (5)	1.000
Impaired consciousness *	16 (13)	4 (10)	12 (14)	0.535
Hypoxemia †	95 (75)	39 (98)	56 (65)	<0.001
Mechanical ventilation †	18 (14)	17 (43)	1 (1)	<0.001
Co-infection with CMV †	18 (14)	7 (18)	11 (13)	0.482

BMI: body mass index, CMV: cytomegalovirus, PJP: *Pneumocystis jirovecii* pneumonia. Data are presented as the number (%) or median (interquartile range). The chi-square or Fisher’s exact test was conducted for categorical variables, and the *t*-test was performed for continuous variables. * The Barthel index, Charlson comorbidity index, and impaired consciousness were evaluated on admission. † Hypoxemia, mechanical ventilation, and co-infection with CMV were evaluated within 7 days from the initiation of TMP-SMX.

**Table 2:** Clinical characteristics of hemodialysis patients with PJP before and after propensity score matching.

	Unmatched			Matched		
	High-Dose TMP-SMX Group (n = 63)	Low-Dose TMP-SMX Group (n = 63)	*p*-Value	High-Dose TMP-SMX Group (n = 36)	Low-Dose TMP-SMX Group (n = 36)	*p*-Value
Sex: female	21 (33)	18 (29)	0.563	12 (33)	15 (42)	0.466
Age, years	71.0 (64.0–76.0)	73.0 (63.0–79.0)	0.875	72.5 (64.3–77.8)	72.0 (68.3–77.8)	0.874
BMI, kg/m^2^	20.3 (18.7–22.3)	22.7 (18.9–25.2)	0.014	20.5 (18.7–22.6)	20.9 (16.7–24.2)	0.921
Missing data	0 (0)	4 (6)	1.000	0 (0)	1 (3)	1.000
Weight	51.4 (46.4–56.0)	56.3 (45.4–67.0)	0.025	51.4 (43.7–59.2)	51.8 (40.9–61.1)	0.932
Barthel index	95.0 (42.5–100.0)	65.0 (21.3–100.0)	0.241	80.0 (26.3–100.0)	85.0 (22.5–100.0)	0.792
0–24	13 (21)	12 (19)	0.823	8 (22)	7 (19)	0.772
25–49	2 (3)	2 (3)	1.000	1 (3)	1 (3)	1.000
50–79	5 (8)	13 (21)	0.049	5 (14)	5 (14)	1.000
80–100	37 (59)	21 (33)	0.005	18 (50)	16 (44)	0.637
Missing data	6 (10)	15 (24)	0.037	4 (11)	7 (19)	0.331
Smoker	25 (40)	22 (35)	0.852	12 (33)	10 (28)	0.801
Missing data	5 (8)	14 (22)	0.031	2 (6)	10 (28)	0.022
Charlson comorbidity index *	2.0 (1.0–3.0)	2.0 (1.0–3.0)	0.480	2.0 (1.0–3.0)	2.0 (1.0–3.0)	0.386
0	4 (6)	6 (10)	0.512	2 (6)	2 (6)	1.000
1–2	35 (56)	34 (54)	0.858	20 (56)	21 (58)	0.812
3–4	16 (25)	19 (30)	0.551	8 (22)	11 (31)	0.424
5–6	3 (5)	3 (5)	1.000	2 (6)	1 (3)	0.563
7–9	5 (8)	1 (2)	0.131	4 (11)	1 (3)	0.197
Impaired consciousness *	5 (8)	11 (18)	0.117	5 (14)	5 (14)	1.000
Hypoxemia †	49 (77)	46 (73)	0.535	28 (78)	26 (72)	0.587
Mechanical ventilation †	11 (18)	7 (11)	0.312	3 (8)	5 (14)	0.458
Co-infection with CMV †	10 (16)	8 (13)	0.611	5 (14)	4 (11)	0.722
Corticosteroid †	60 (95)	54 (86)	0.082	34 (94)	34 (94)	1.000
Steroid pulse †‡	22 (35)	7 (11)	0.002	7 (19)	7 (19)	1.000
Combination therapy	7 (11)	3 (5)	0.200	3 (8)	2 (6)	0.645
Atovaquone	4 (6)	1 (2)	0.205	1 (3)	1 (3)	1.000
Pentamidine	3 (5)	1 (2)	0.333	2 (6)	1 (3)	0.563
Caspofungin	0 (0)	1 (2)	1.000	0 (0)	0 (0)	1.000

BMI: body mass index, CMV: cytomegalovirus, PJP: *Pneumocystis jirovecii* pneumonia, TMP-SMX: trimethoprim–sulfamethoxazole. Data are presented as the number (%) or median (interquartile range). Logistic regression analysis was performed. * Barthel index, Charlson comorbidity index, and impaired consciousness were evaluated on admission. † Hypoxemia, mechanical ventilation, co-infection with CMV, and administration of corticosteroid or steroid pulse were evaluated within 7 days from the initiation of TMP-SMX. ‡ Maximum daily corticosteroid dose ≥100 mg/day of methylprednisolone equivalent.

**Table 3:** The TMP-SMX tolerance, adverse events, and 90-day death rate in the hospital before and after propensity score matching.

	Unmatched			Matched		
	High-Dose TMP-SMX Group (n = 63)	Low-Dose TMP-SMX Group (n = 63)	*p*-Value	High-Dose TMP-SMX Group (n = 36)	Low-Dose TMP-SMX Group (n = 36)	*p*-Value
Dose of TMP, mg/kg/day	8.18 (6.96–9.12)	4.36 (3.16–5.29)	<0.001	8.50 (7.18–9.55)	4.28 (3.25–4.98)	<0.001
Dose of SMX, mg/kg/day	40.9 (34.8–45.6)	21.8 (15.8–26.4)	<0.001	42.5 (35.9–47.8)	21.4 (16.3–24.9)	<0.001
Duration of TMP-SMX, days	16.0 (12.0–21.0)	17.0 (12.0–21.0)	0.557	16.0 (12.0–20.8)	14.0 (12.0–19.8)	0.587
Duration of PJP treatment including other drugs, days	17.0 (13.0–22.0)	18.0 (12.0–21.0)	0.855	17.0 (13.3–22.0)	16.0 (12.0–21.0)	1.000
Dose reduction of TMP-SMX	8 (13)	6 (10)	0.571	4 (11)	2 (6)	0.674
Switching to other treatments	15 (24)	9 (14)	0.173	7 (19)	8 (22)	0.772
Atovaquone	11 (18)	5 (8)	0.108	6 (17)	4 (11)	0.496
Pentamidine	6 (10)	6 (10)	1.000	3 (8)	6 (17)	0.478
Adverse event *	29 (46)	26 (41)	0.590	17 (47)	16 (44)	0.813
Hypoglycemia †	24 (38)	16 (25)	0.126	15 (42)	11 (31)	0.326
Hyponatremia †	8 (13)	12 (19)	0.329	4 (11)	7 (19)	0.326
Thrombocytopenia †	7 (11)	2 (3)	0.164	2 (6)	1 (3)	1.000
90-day deaths in hospital	26 (41)	14 (22)	0.022	13 (36)	10 (28)	0.448

PJP: *Pneumocystis jirovecii* pneumonia, TMP-SMX: trimethoprim–sulfamethoxazole. Data are presented as the number (%) or median (interquartile range). The chi-square or Fisher’s exact test was conducted for categorical variables, and the *t*-test was performed for continuous variables. * Overlap allowed. † Hypoglycemia was determined by administration of hypertonic sugar solution, hyponatremia by administration of hypertonic sodium solution, and thrombocytopenia by platelet transfusion.

**Table 4:** Characteristics of hemodialysis patients with PJP in the survivor and nonsurvivor groups (within 90 days) after propensity score matching.

	Nonsurvivor (n = 23)	Survivor (n = 49)	HR	*p*-Value
Sex: female	8 (35)	19 (39)	0.983 (0.412–2.348)	0.970
Age, years	74.0 (70.0–79.0)	71.0 (63.5–77.5)	1.022 (0.978–1.068)	0.327
BMI, kg/m^2^	21.4 (18.7–22.7)	20.4 (17.6–23.5)	0.997 (0.882–1.128)	0.968
Weight	53.2 (43.3–59.8)	51.2 (41.9–59.7)	0.995 (0.962–1.030)	0.796
Barthel index	55.5 (12.5–85.0)	85.0 (56.3–100.0)	0.991 (0.979–1.003)	0.126
Smoker	5 (22)	17 (35)	0.471 (0.156–1.417)	0.180
Charlson comorbidity index *	2.0 (1.0–3.0)	2.0 (1.0–3.0)	1.004 (0.823–1.226)	0.966
Impaired consciousness *	4 (17)	6 (12)	1.862 (0.615–5.644)	0.272
Hypoxemia †	23 (100)	31 (63)	31.776 (0.494–2043.814)	0.104
Mechanical ventilation †	8 (35)	0 (0)	7.859 (3.182–19.410)	<0.001
Co-infection with CMV †	3 (13)	6 (12)	0.782 (0.228–2.678)	0.695
High dose TMP-SMX	13 (57)	23 (47)	1.358 (0.575–3.210)	0.485
Corticosteroid †	21 (91)	47 (96)	1.031 (0.237–4.485)	0.968
Steroid pulse †‡	6 (26)	8 (16)	1.507 (0.587–3.868)	0.394

BMI: body mass index, CMV: cytomegalovirus, HR: hazard ratio, PJP: *Pneumocystis jirovecii* pneumonia, TMP-SMX: trimethoprim–sulfamethoxazole. Data are presented as the number (%) or median (interquartile range). Univariate analyses were performed using Cox hazard analysis to determine the association between the in-hospital deaths within 90 days and other factors. * Barthel index, Charlson comorbidity index, and impaired consciousness were evaluated on admission. † Hypoxemia, mechanical ventilation, co-infection with CMV, and administration of corticosteroid or steroid pulse were evaluated within 7 days from the initiation of TMP-SMX. ‡ Maximum daily corticosteroid dose ≥100 mg/day of methylprednisolone equivalent.

## Data Availability

The datasets generated and analyzed during the current study are not publicly available due to a license agreement with Tokyo Medical and Dental University Graduate School. However, they are available from the corresponding author upon reasonable request.

## Supplementary Materials

The following supporting information can be downloaded at: https://www.mdpi.com/article/10.3390/jcm13185463/s1, Figure S1: ROC curve for propensity score; Table S1: Co-treatment of hemodialysis patients with PJP in the survivor and non-survivor groups (within 90 days of treatment).
