# Supplementary material for: Comparison of High-Dose versus Low-Dose Trimethoprim–Sulfamethoxazole for Treating Pneumocystis jirovecii Pneumonia among Hemodialysis Patients: A Nationwide Database Study in Japan
Source: J Clin Med. 2024 Sep 14;13(18):5463. doi: 10.3390/jcm13185463 (PMC11432082; doi:10.3390/jcm13185463)

Supplementary file

# Comparison of High-Dose Versus Low-Dose Trimethoprim–Sulfamethoxazole for Treating *Pneumocystis jirovecii* Pneumonia among Hemodialysis Patients: A Nationwide Database Study in Japan

**Hisayuki Shuto [1], Shota Omori [1], Kazufumi Hiramatsu [1,2], Jun-ichi Kadota [1], Kiyohide Fushimi [3] and Kosaku Komiya [1,2,*]**

[1] Respiratory Medicine and Infectious Diseases, Faculty of Medicine, Oita University, 1-1 Idaigaoka, Hasama-machi, Yufu 879-5593, Oita, Japan; shuto0326@oita-u.ac.jp (H.S.); s-omori@oita-u.ac.jp (S.O.); hiramats@oita-u.ac.jp (K.H.); kadota@oita-u.ac.jp (J.-i.K.)

[2] Research Center for Global and Local Infectious Diseases, Faculty of Medicine, Oita University, 1-1 Idaigaoka, Hasama-machi, Yufu 879-5593, Oita, Japan

[3] Department of Health Policy and Informatics, Tokyo Medical and Dental University Graduate School, 1-5-45 Yushima, Bunkyo-ku 113-8519, Tokyo, Japan; kfushimi.hci@tmd.ac.jp

* Correspondence: komiyakh1@oita-u.ac.jp; Tel.: +81-97-586-5804; Fax: +81-97-549-4245

**Supplementary Table S1. Co-treatment of hemodialysis patients with PJP in the survivor and non-survivor groups (within 90 days of treatment).**

| | All patients (n = 126) | Non-survivor (n = 40) | Survivor (n = 86) | *P* Value |
|---|---|---|---|---|
| Corticosteroid* | 114 (91) | 38 (95) | 76 (88) | 0.336 |
| Steroid pulse*† | 29 (23) | 13 (33) | 16 (18) | 0.085 |
| High dose TMP-SMX | 63 (50) | 26 (65) | 37 (43) | 0.022 |
| Dosage of TMP | 5.74 (4.33-8.18) | 7.03 (4.70-8.72) | 5.58 (4.17-7.59) | 0.029 |
| Dosage of SMX | 28.7 (21.6-40.9) | 35.1 (23.5-43.6) | 27.9 (20.8-38.0) | 0.029 |
| Combination therapy | 10 (8) | 3 (8) | 7 (8) | 1.000 |
| Atovaquone | 5 (4) | 0 (0) | 5 (6) | 0.177 |
| Pentamidine | 4 (3) | 3 (8) | 1 (1) | 0.094 |
| Caspofungin | 1 (1) | 0 (0) | 1 (1) | 1.000 |

PJP: *Pneumocystis jirovecii* pneumonia, TMP-SMX: trimethoprim-sulfamethoxazole.
Data are presented as the number (%) or median (interquartile range). The Chi-square or Fisher's exact test was conducted for categorical variables, and the t-test was performed for continuous variables.
* Administration of corticosteroid or steroid pulse were evaluated within 7 days from the initiation of TMP-SMX.
† Maximum daily corticosteroid dose ≥100 mg/day of methylprednisolone equivalent.

**Supplementary Figure S1. ROC curve for propensity score**

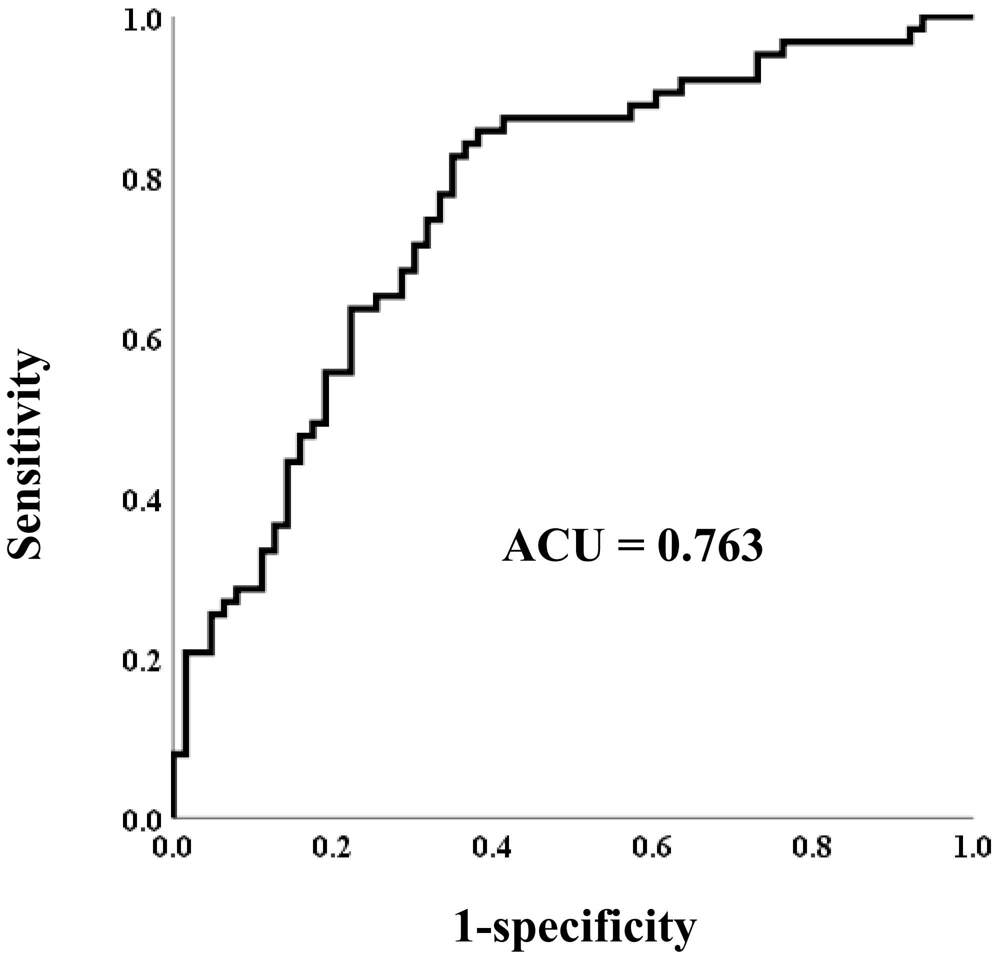

Supplement: Supplementary file 1 [file jcm-13-05463-s001.zip › jcm-3177738-supplementary.pdf]
